# X-ray Microanalysis of Elemental Composition of *Vitis sylvestris* Pollen Grains

**DOI:** 10.3390/plants13162338

**Published:** 2024-08-22

**Authors:** Katarina Lukšić, Ana Mucalo, Luka Marinov, Maja Ozretić Zoković, Zorica Ranković-Vasić, Dragan Nikolić, Goran Zdunić

**Affiliations:** 1 Institute for Adriatic Crops and Karst Reclamation, 21000 Split, Croatia; katarina.luksic@krs.hr (K.L.); ana.mucalo@krs.hr (A.M.); luka.marinov@krs.hr (L.M.); maja.ozretic@krs.hr (M.O.Z.); 2 Centre of Excellence for Biodiversity and Molecular Plant Breeding (CoE CroP-BioDiv), Svetošimunska Cesta 25, 10000 Zagreb, Croatia; 3 Faculty of Agriculture, University of Belgrade, 11080 Belgrade, Serbia; zoricarv@agrif.bg.ac.rs (Z.R.-V.); nikolicd@agrif.bg.ac.rs (D.N.)

**Keywords:** *Vitis vinifera* subsp. *sylvestris*, pollen surface, exine, chemical elements, SEM-EDX

## Abstract

The flowering and fruit set of grapevines are determined by many morphological, physiological, and environmental factors. Although the elemental composition of pollen grains plays a crucial role in the fruit set, there is still a considerable gap in our knowledge. To date, no study has been conducted on the elemental composition of *Vitis vinifera* subsp. *sylvestris* (hereafter *V. sylvestris*) pollen grains. The aim of this work was to investigate the elemental composition of pollen grains of *V. sylvestris* using scanning electron microscopy with energy dispersive X-ray spectroscopy (SEM-EDX). The pollen grains of ten *V. sylvestris* individuals (eight male and two female) and one hermaphrodite cultivar ‘Plavac mali crni’ were analyzed. SEM-EDX analysis revealed the presence of eight elements (carbon—C, oxygen—O, magnesium—Mg, phosphorus—P, potassium—K, calcium—Ca, molybdenum—Mo, and aluminum—Al) in the pollen grains. Interestingly, aluminum was detected exclusively in the pollen of the cultivated grape cultivar ‘Plavac mali crni’, while it was not present in the genotypes of *V. sylvestris*. No significant differences between genotypes were found for oxygen and phosphorus, while significant differences were found for other elements. Pollen dimorphism was not associated with differences in element composition, although principal component analysis separated the genotypes into two distinct groups, with two female individuals (Pak10 and Pak12) and one male (Im19) tending to form separate clusters. This study is the first report on the elemental composition of pollen grains of *V. sylvestris* genotypes and provides valuable insights for further studies on pollen functionality.

## 1. Introduction

Flower development and fruit set are of utmost importance for grapevine production, which is reflected in their formation over two seasons [1,2]. Grapevine flowering occurs at a time when a marked switch in nutritional allocation takes place [2]. Mineral nutrition involves one of the most complex interactions affecting grapevine fertility. Carbohydrates are the main source of energy and play a role in flowering and determining vine yield potential [1,2].

There is a list of nutrients that are important for grapevine flowering, with calcium, zinc, boron, and molybdenum being particularly involved [2,3]. The presence of normal and small berries in the bunch (‘millerandage’) is associated with a deficiency of zinc and boron [4]. Molybdenum has been found in relatively high concentrations in pollen compared to other parts of the vine [5]; its deficiency causes a low pollen number and viability with reduced enzymatic activity [6]. Calcium is necessary for pollen tube growth and germination [3], with several calcium-binding zones associated with the protein DEX1 in the pollen wall [7].

Pollen plays a fundamental role in grapevine reproduction. In wild grapevine (*V*. *sylvestris*), the dimorphism of pollen is well documented, differing mainly in their functionality and yield potential. Sterile pollen grains (inaperturate) are found in functionally female flowers, while fertile pollen grains (trizonocolporate) are found in hermaphroditic flowers of cultivated grapevine and functionally male flowers of *V. sylvestris* [8,9].

The pollen grain is surrounded by a multilayered outer surface, the pollen wall [10], which consists of an outer part exine and an inner part intine [11]. The exine is structurally more complex than any other plant cell wall [7,10], composed of sporopollenin, a biopolymer with very different amounts of hydrogen and oxygen in the general formula C_90_ H_142_ O_36_ [11]. The development of the pollen wall mainly involves genes and proteins that regulate lipid and polysaccharide metabolism [10]. The outer parts of the pollen are composed of fatty acids and phenolic compounds, while the inner content is related to polysaccharide metabolism. A lack of expression of lipid metabolism genes for sporopollenin biosynthesis can lead to male sterility [10]. In the *Arabidopsis* male sterility mutant, the endintine was thicker and the exintine was thinner. Poor nutrition by the tapetum cells (precursors of the pollen wall) was associated with low pollen fertility in cultivated grapevines [9,12].

Electron microscopy has led to a major breakthrough in palynology in the last ~60 years [11]. Energy dispersive X-ray spectroscopy (EDX), combined with scanning electron microscopy (SEM), enables the detection of the elemental composition of samples [13], allowing analysis of surface depth of one to two micrometers [14]. This corresponds to the exine thickness (1 to 1.6 µm) in *Vitis vinifera* L. [9,15]. The analysis of pollen exines by SEM-EDX was previously not considered a suitable method due to limitations such as shallow depth, detection of elements with an atomic number of less than 10 [13,16,17], or variations in surface geometry [16]. Recently, however, this method has shown promise for nanotechnology to detect elemental deposits of disease agents, pollution, in personalized medicine, etc. [17].

The elemental composition of pollen has been analyzed in an increasing number of plant species using the SEM-EDX technique. Higher silicon and calcium content found in the exine of *Impatiens sultanii* was related to a thicker exine, ensuring greater pollen resistance [16]. Bucsek et al. found six elements: magnesium, phosphorus, sulfur, chlorine, potassium, and calcium as the main components of pollen in apple, pear, cherry, sour cherry, plum, almond, apricot, peach, and quince [18]. Duque et al. reported that mineral dust alters the elemental composition of air-borne pollen surface, emphasizing the influence of the sea in coastal regions [19]. Acma and Mendez recorded higher levels of potassium, sulfur, magnesium, and aluminum in the pollen of four native ginger species, with aluminum being associated with inhibition of pollen tube growth [20].

Studies on *Vitis* spp. pollen have recently gained attention [9,15,21], but data on the chemical composition of *Vitis vinifera* L. pollen are scarce. Kidman et al. investigated the nutrient composition of Shiraz grapevine pollen using inductively coupled plasma optical emission spectrometry (ICP-OES) for nutrients involved in pollination and fertilization (boron, zinc, calcium, and molybdenum) [3]. The elemental composition of grapevine pollen analyzed with SEM-EDX was not found. However, interesting studies have been carried out with EDX in grapevine to characterize calcium oxalate phytolith crystals, which play a role in strengthening cell walls or serve as carbon stores [22].

The wild grapevine (*V. sylvestris*) in Croatia is being studied in detail as part of various projects and initiatives. Knowledge about the current distribution, morphological, and genetic traits has been collected and published in a dozen papers, including a detailed characterization of pollen microstructure [23,24]. Due to the functional dimorphism observed in pollen of female vs. hermaphrodite and male individuals, more detailed studies are needed to better understand the morphology and function of grapevine pollen. The aim of this work was to characterize the elemental content of the pollen wall of wild grapevine (*V*. *sylvestris*) using scanning electron microscopy (SEM) in combination with energy dispersive X-ray spectroscopy (EDX). The results will contribute to a better understanding of the elemental composition of wild grapevine pollen, palaeoecology, and in a broader context, functionality of the grapevine flower.

## 2. Materials and Methods

### 2.1. Sampling Sites and Plant Material

**Figure 1:**
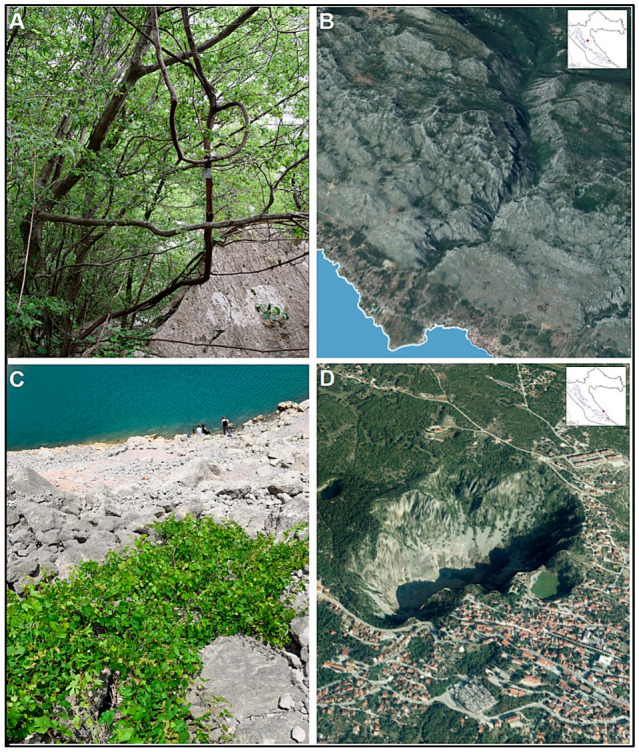
Two natural populations, NP Paklenica (**A**,**B**) and Imotski Blue Lake (**C**,**D**), represent the main growth and habitat differences of *Vitis vinifera* subsp. *sylvestris* along the eastern Adriatic coast in the northern Mediterranean region.

Pollen from flowers of 10 *V. sylvestris* individuals and one *V. vinifera* cultivar (cv.) ‘Plavac mali crni’ (PMC) was sampled at full bloom in 2018. *V. sylvestris* originated from two natural sites: Paklenica National Park and Blue Lake in Imotski. Representative set of two female and eight male individuals was chosen, portraying the greater occurrence of male compared to female individuals at natural sites [24]. PMC originated from a field cultivation in the grapevine germplasm collection in Split at the Institute for Adriatic Crops and Karst Reclamation (IAC, HRV048), planted in 2006. Both compartments are growing in rain-fed environments (no artificial irrigation systems). *V. sylvestris* locations are shown in Figure 1, and are characterized by a typical karst relief, an arid Mediterranean climate, and nutrient-poor soils [25]. Paklenica is among the best preserved and the coldest mountainous and humid sites with an abundance of supporting plant species for vines, whereas the Imotski population near the urban center is characterized by poor accompanying flora and erosion-prone, very shallow soils with pronounced fluctuations in the water level in the lake (Figure 1).

Two inflorescences per genotype were sampled during full bloom, from 2 June to 7 June (~50% open flowers) at natural wild grape sites and in vineyards for cultivated grapevines. Inflorescences were placed in 50 mL polypropylene tubes, dried with silica-gel beads, and stored at −20 °C, until scanning electron microscopy analyses.

### 2.2. Scanning Electron Microscopy and Elemental Composition of Pollen

The surface of silica gel-dried pollen samples of *Vitis vinifera* L. was analyzed in the laboratory of the Faculty of Agriculture (Belgrade-Zemun, Serbia) using a scanning electron microscope (SEM) JEOL JSM-6390LV (Tokyo, Japan), as previously described [24,26,27,28].

The anthers were isolated from the flowers, placed in small vials and stored at 3–5 °C in a silica gel desiccator. After dehydration, the pollen was applied with a fine brush to aluminum sticks, covered with double-sided transparent tape for analysis. The samples were then coated with a 0.02 μm thick layer of gold using a sputter coater (BAL-TEC SCD 005, Capovani Brothers Inc., Scotia, NY, USA). Initially, ~30 pollen grains per genotype were analyzed using SEM to reveal microstructure of pollen grains [24].

For the elemental composition in this study, a single pollen grain per genotype was selected and three different sections on the surface of a pollen grain were analyzed (marked as ‘boxes’ in Figure S1).

The elemental composition of the surface of pollen was determined by EDX. EDX enables the evaluation of the material. When the sample is exposed to the electron beam in the SEM, it interacts with the beam and produces characteristic X-rays. Each element has a specific emission of X-rays. EDX was performed using an X-MaxN X-ray detector (Oxford Instruments, Abingdon, UK) with the following analysis parameters: HV (high voltage) examination mode, 15 Kv accelerating voltage and 10 mm WD (working distance), signal SEI (Secondary Electron Imaging, JEOL JSM-6390LV, Tokyo, Japan), and magnification 3000×. The Aztec 2.2 software was used for elemental mapping.

**Figure 2:**
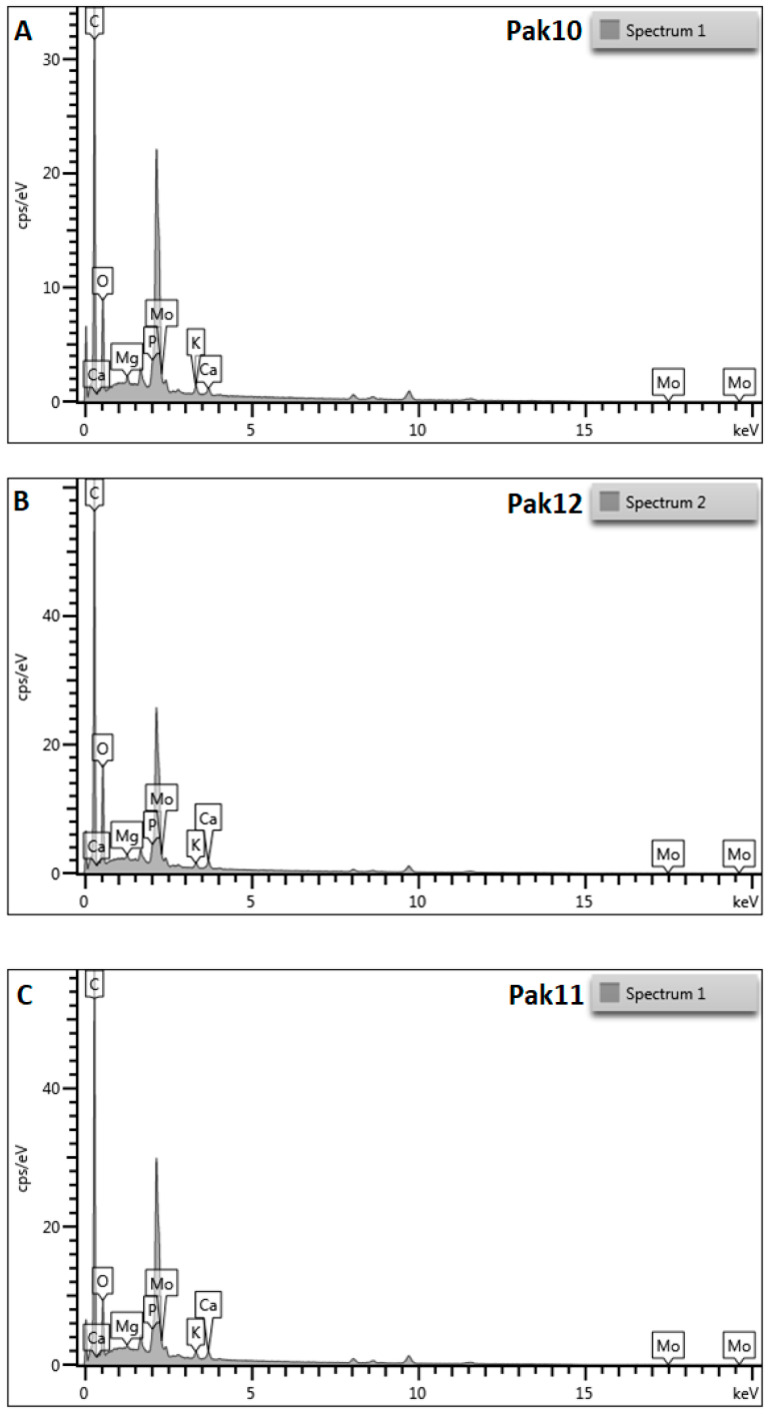

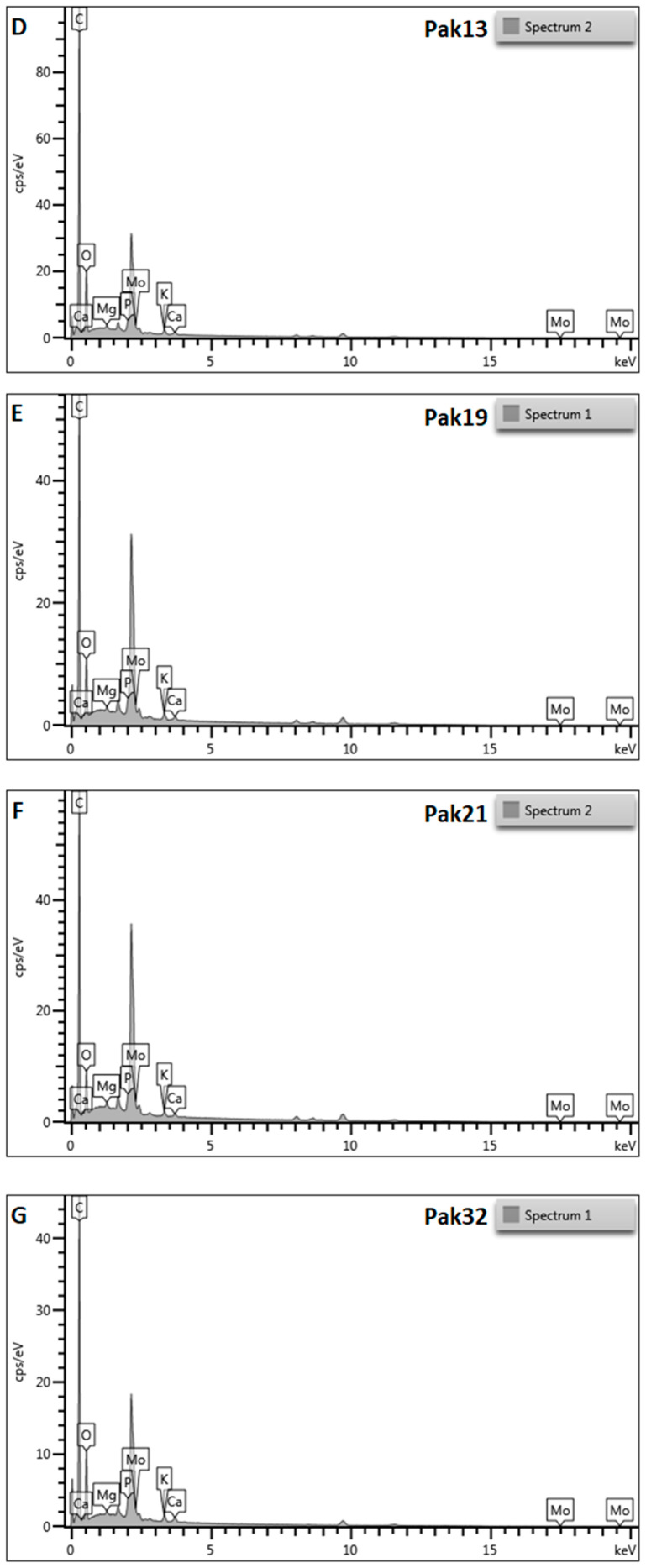

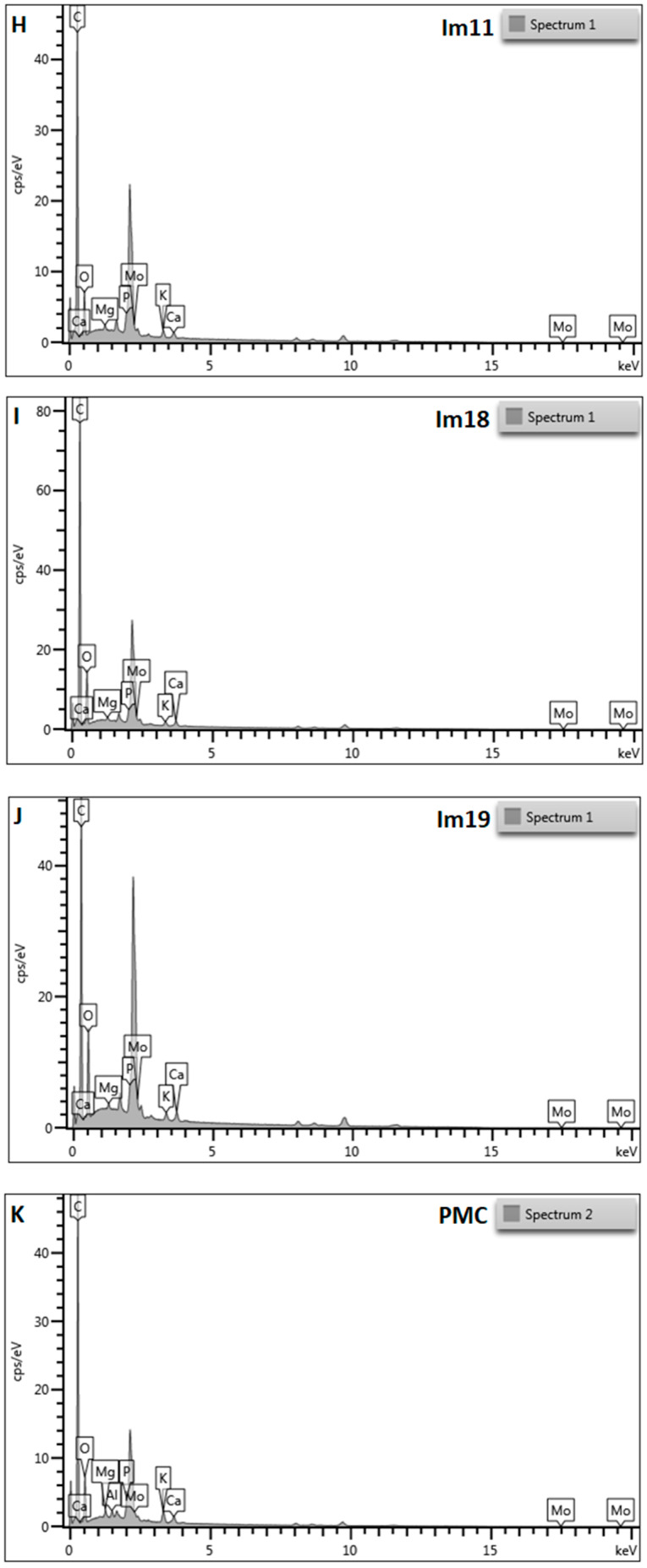
SEM-EDX analysis of the elemental composition of pollen grains from ten *Vitis vinifera* subsp. *sylvestris* two female (**A**,**B**), eight male (**C**–**J**) and one cultivated *Vitis vinifera* cv. ‘Plavac mali crni’ (**K**).

Table 1 shows the mean values for the elemental composition determined from the entire scanned area (all three sections). Results for elemental composition shown in Figure 2, correspond to the yellow highlighted ‘boxes’ in Figure S1.

### 2.3. Statistical Analysis

Data were statistically analyzed using one-way analysis of variance (ANOVA) with HSD Tukey post hoc to test for differences between genotypes; significant differences were at values *p* ≤ 0.05. Genotypes were classified into groups based on elemental composition (except aluminum) using principal component analysis (PCA). The ANOVA and Tukey test were performed in STATISTICA v. 14.0 (TIBCO Software Inc., Palo Alto, CA, USA) while for PCA, R Studio 2023.03.0 + 386 was used with FactoMineR and factoextra packages [29,30].

## 3. Results

### 3.1. Elemental Composition of the Pollen of Vitis vinifera L.

The elemental composition of pollen from 10 *V. sylvestris* and one *V. vinifera* genotypes analyzed by SEM-EDX revealed the presence of a total of eight elements: carbon, oxygen, magnesium, phosphorus, potassium, calcium, molybdenum, and aluminum (Table 1, Figure 2). The most abundant element was carbon with a percentage of 70.57 to 77.82%, followed by oxygen with a percentage of about 18% in the genotypes analyzed. Phosphorus, potassium, magnesium, and calcium as macronutrients were detected in a low proportion of about 1%. The micronutrient molybdenum was detected in proportions between 1.89 and 3.92%. Significant differences between individuals were found in the content of five elements (carbon, magnesium, potassium, calcium, and molybdenum), while the content of oxygen and phosphorus showed no significant differences in this study. Aluminum was detected only in the pollen grains of *V. vinifera* cv. ‘Plavac mali crni’ (PMC).

The carbon content differed significantly only among three individuals of the Imotski population. The males Im11 (77.82%) and Im18 (77.63%) had a significantly higher carbon content than the male Im19 (70.57%) (Table 1). Molybdenum was significantly higher in two individuals from the Paklenica population (female Pak10 (3.85%) and male Pak21 (3.92%)) than in male Im18 (1.89%). Potassium content differed only between three individuals, where cv. PMC (1.53%) had significantly higher content than two male individuals (Pak11 (0.77%) and Im18 (0.56%)). Calcium content was significantly higher in female Pak10 (1.08%) than in male Pak13 (0.58%). The male Im19 (1.29%) had a higher calcium content than three male individuals from the Paklenica population (Pak19 (0.74%), Pak21 (0.66%) and Pak13 (0.58%)). The male individuals Im19 and Pak13 differed the most in calcium content. The magnesium content of cv. PMC (0.40%) and female Pak10 (0.38%) was significantly higher than that of male individuals Pak32 (0.25%), Im18 (0.19%), and Pak11 (0.17%). Cv. PMC and Pak10 together with male individuals Pak19, Pak21, and Im11 had significantly higher magnesium content than the two male individuals Im18 and Pak11. The largest significant difference in magnesium content was observed between female Pak10 and male Pak11—two neighbouring individuals. In this study, there was no substantial difference in the elemental composition of the pollen surface between *V. sylvestris* set and cv. PMC, with the exception of aluminum content.

### 3.2. Principal Component Analysis (PCA) Based on Elemental Pollen Composition 

**Figure 3:**
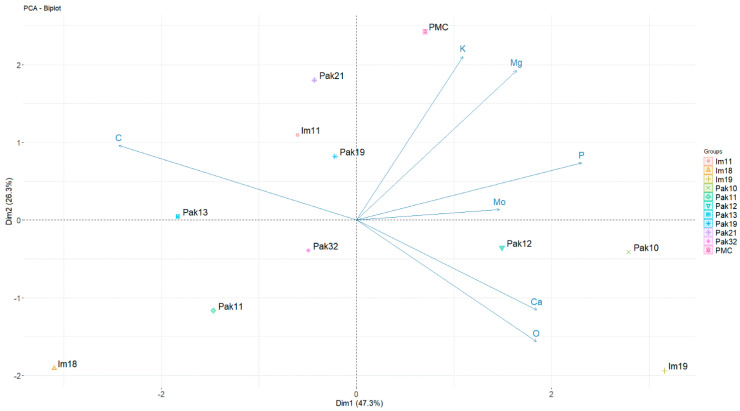
Principal component analysis (PCA) of ten *Vitis vinifera* subsp. *sylvestris* and one *Vitis vinifera* cv. PMC, based on the SEM-EDX elemental composition of the pollen surface. The grouping in the PCA plot based on seven elements: Carbon (C), Oxygen (O), Magnesium (Mg), Phosphorus (P), Potassium (K), Calcium (Ca) and Molybdenum (Mo) in two female (Pak10 and Pak12) and eight male *Vitis vinifera* subsp. *sylvestris* individuals (Pak11, Pak13, Pak19, Pak21, Pak32, Im11, Im18 and Im19) and one *Vitis vinifera* cv. ‘Plavac mali crni’ (PMC).

Principal component analysis (PCA) was applied to find patterns and explain the variance of elemental composition in the analyzed *V. vinifera* set (without aluminum) with two principal components (DMs). PCA explained 73.6% of the variance, with DM1 explaining 47.3% of the total diversity (Figure 3). Grouping the individuals along DM1 resulted in a separation into three groups. On the left side of the plot, male *V. sylvestris* individuals from both wild populations grouped together. None of the elements, with the exception of carbon, showed a positive correlation with this grouping, suggesting a poorer elemental composition in the male *V. sylvestris* individuals. Both female *V. sylvestris* and one male Im19 individual were grouped in the lower right of the plot. Exceptional grouping of the male Im19 with the female individuals was mainly determined by the strong positive correlation with calcium content, which was highest in Im19. The highest calcium content in this sample set, but also the highest oxygen content, could be interesting in the case of Im19, as this individual is closest to the surface of Imotski’s Blue Lake which experiences pronounced water level fluctuations. In contrast, *V. vinifera* cv. PMC was separated from the samples of *V. sylvestris* in the upper right part of the plot, due to the higher content of elements related to the quality of grapevine, such as potassium, magnesium (or phosphorus).

For DM2, accounting for 26.3% of the variation, the individuals in the lower part of the biplot differed from those in the upper part due to differences in the composition of the elements magnesium, potassium, and calcium, which exhibit the most significant differences among individuals in this sample set (Table 1).

PCA grouping based on elemental composition resulted in separation by quality and quantity of elemental composition as well as separation by flower type.

## 4. Discussion

The elemental composition of the pollen grains of ten *V. sylvestris* and one *V. vinifera* genotype was determined by SEM-EDX spectroscopy.

Significant differences were found for five of eight elements, with carbon accounting for the largest proportion (71–78%). This reflects the fact that pollen grains are generally a rich source of carbohydrates and germinate in a ‘sucrose’ environment [1,9,31]. A significant difference in carbon content was observed only among the male individuals of the Imotski population. The individual Im19 (Figure 1C) had the lowest carbon content, while the other two individuals had a significantly higher carbon content compared to Im19. The variations in carbon content might be related to the fluctuations of the water level in the lake during the season, as the plants in Imotski occasionally grow completely submerged [25]. Under such conditions, aquatic or terrestrial flooded plants may exhibit reduced photosynthesis and carbon uptake, and light interception is attenuated with depth [32]. 

Calcium is involved in the formation, composition, and stabilization of cell walls [33]. It provides rigidity to the pollen tube wall by binding to pectin carboxyl groups and is a key factor for proper pollen germination and tube growth [34,35,36,37,38]. The largest difference in calcium content between the two populations, observed in two male individuals (Im19 vs. Pak13), indicates the differences in contrasting growth conditions rather than nutrient availability or flower type. Within the Paklenica population, a significant difference in calcium content was observed only between female Pak10 (higher content) and male Pak13 (lower content). In rice, fertile pollen accumulated calcium precipitates on the surface of the pollen grains and in the anther walls, whereas in sterile pollen, calcium exudates were detected in the middle layer and not in the tapetum cells as in fertile pollen. Sterile anthers had a special cell wall between the tapetum and middle layer, indicating a different pattern of calcium accumulation and poor pollen wall formation in sterile anthers. In this way, irregularities in calcium distribution contribute to failure of pollen development [39]. The PCA grouping of female individuals, which is mainly determined by calcium content (Figure 3), is consistent with this observation. Calcium content is related to a thicker exine, which increases the resistance of pollen to harsh conditions [16], strengthens cell walls [22] and, in the case of female flowers with sterile pollen, is probably part of the excessive deposition of material on the surface of the pollen wall due to a lack of apertures [9]. Over-accumulation of calcium on the cell membranes can lead to abnormal microspore development [40]. Calcium has a particularly strong affinity to bind to the phosphate groups of all phospholipids [41]. The pollen wall contains the highest calcium content in pollen grains [40], and the specific gene DEX1 is associated with calcium binding sites in the pollen wall [7].

The third most abundant element detected in the pollen wall was molybdenum (~2 to 4%). A significant difference in molybdenum was evident among three individuals, where two individuals (female and male) from Paklenica had a molybdenum content twice as high as the male Im18 from the Imotski population. The significantly lower content in Im18 could be related to occasional water fluctuations, uneven soil depth (shallow) due to erosion [25], or poorer leaf litter and organic matter to which molybdenum binds [42,43]: in particular, water fluctuations may correlate, as molybdenum is soluble in water and oxyic systems and is present in deeper soils as it is prone to leaching [42,44]. Alternatively, but less likely, lower molybdenum content could be due to surface corrosion processes on pollen, as biodegradable and bioresorbable properties have recently been reported for this element [45,46,47].

The pollen of the cultivated variety (PMC) was mainly characterized by the content of potassium and magnesium, two elements displaying a strong positive correlation in the PCA plot (Figure 3), but have an overall low proportion (less than 1%) of the pollen surface. Potassium is often referred to as the ‘element of quality’ [48,49]. Pollen from hermaphrodite and male flowers is usually normal and fertile; however, the pollination outcome in wild grapevines is usually drastically low compared to cultivated grapevines [50,51]. In barley pollen, the distribution of potassium was different during the developmental stages, with higher levels observed in the pollen wall in the early stages of development, while in later stages, higher levels were observed around the aperture area [52]. It could be that the lower potassium content in two male individuals is due to a shift from the pollen wall to the aperture area, which usually have a different exine construction [11] appearing lower in surface EDX analysis; or to poorer availability and uptake of potassium in these individuals.

Magnesium content was significantly higher in cv. PMC and female individual Pak10 compared to three male individuals, and interestingly, a substantial difference was found between two neighboring individuals (female/high vs. male/low).

Aluminum was found only in cv. PMC, suggesting surface airborne aluminum deposition, i.e., pollution [53], probably related to the use of aluminum-containing pesticides in commercial viticulture, unlike the natural habitat of *V. sylvestris*, where no spraying is done at all. There is little data on aluminum pollen contamination, such as the one report of high aluminum contamination in bee pollen [54]. The European Food Safety Authority (EFSA) risk assessment indicates that contamination with aluminum in pollen and bee products is unlikely as it is not systemic and therefore unlikely to be transferred to plant tissue [55].

Interestingly, no zinc was detected on the pollen surface by EDX, although zinc is essential for pollen development and its deficiency can lead to coulure or millerandage in grapevine [3].

EDX analysis has objective limitations, such as low depth resolution, the presence of elements in only a limited scanned area, overlap in X-ray signal, signal loss for some elements, or errors due to sample preparation [14,56]. Sample preparation can lead to alterations such as dehydration of the pollen cytoplasm, which reduces its volume and leads to changes in the shape and size of the pollen wall (harmomegathy) [7,57]. Nevertheless, EDX proved to be very informative for the characterization of *V. sylvestris* and *V. vinifera* pollen, providing an insight into the nutritional status of *V. vinifera* L. pollen grains.

## 5. Conclusions

The elemental composition of the surface of the pollen grains of *V. sylvestris*, analyzed by the SEM-EDX method, revealed the presence of eight elements, with five exhibiting significant differences between individuals. The results obtained with this sample set and the statistical analysis do not allow a clear distinction between individuals of *V. sylvestris* based on their flower type or population of origin. There were no substantial differences in elemental composition between *V. sylvestris* and *V. vinifera*; consistent with previous data on the pollen microstructure of *V. vinifera* L., confirming the functional nature of dimorphism in grapevine pollen. Individual significant changes detected for some elements indicate peculiarities related to the nutritional status of the plant and their habitat. The SEM-EDX has proven useful in this study to gain a deeper insight into the nutritional status and requirements of *V. vinifera* L. Further microscopic and genetic studies with a larger number of samples would be interesting to understand the role of nutrients, flowering, and yield formation in grapevine in a specific environment.

## Figures and Tables

**Table 1:** The elemental surface composition of pollen grains of ten *Vitis vinifera* subsp. *sylvestris* genotypes from two natural populations (Paklenica and Imotski): two female (F), eight male (M) individuals, and one hermaphrodite (H) *Vitis vinifera cv.* ‘Plavac mali crni’ from the IAC collection, analyzed by scanning electron microscopy (SEM) and energy dispersive X-ray spectrometry (EDX).

Genotype	Flower	Carbon	Oxygen	Magnesium	Phosphorus	Potassium	Calcium	Molybdenum	Aluminum
Pak10	F	71.67 ± 3.23 ab	20.76 ± 5.35	0.38 ± 0.02 a	1.30 ± 0.18	0.96 ± 0.25 ab	1.08 ± 0.31 bc	3.85 ± 1.51 a	nd
Pak12	F	73.58 ± 3.91 ab	19.96 ± 5.27	0.31 ± 0.06 abc	1.39 ± 0.43	0.97 ± 0.36 ab	1.01 ± 0.15 abc	2.78 ± 0.49 ab	nd
Pak11	M	77.05 ± 1.85 ab	16.93 ± 2.47	0.17 ± 0.03 d	1.05 ± 0.14	0.77 ± 0.13a	0.99 ± 0.05 abc	3.04 ± 0.40 ab	nd
Pak13	M	76.49 ± 2.44 ab	18.43 ± 3.68	0.28 ± 0.04 abcd	0.89 ± 0.28	0.89 ± 0.24 ab	0.58 ± 0.17 a	2.43 ± 0.58 ab	nd
Pak19	M	75.45 ± 1.75 ab	17.79 ± 3.13	0.34 ± 0.04 ab	1.00 ± 0.22	1.05 ± 0.25 ab	0.74 ± 0.09 ab	3.64 ± 0.80 ab	nd
Pak21	M	77.29 ± 0.39 ab	15.57 ± 1.39	0.36 ± 0.07 ab	1.28 ± 0.41	0.92 ± 0.22 ab	0.66 ± 0.13 ab	3.92 ± 0.93 a	nd
Pak32	M	75.51 ± 3.64 ab	18.42 ± 4.18	0.25 ± 0.04 bcd	1.13 ± 0.10	0.92 ± 0.13 ab	0.83 ± 0.12 abc	2.94 ± 0.20 ab	nd
Im11	M	77.82 ± 0.69 a	15.79 ± 1.19	0.33 ± 0.07 ab	1.15 ± 0.11	1.04 ± 0.14 ab	1.03 ± 0.06 abc	2.83 ± 0.35 ab	nd
Im18	M	77.63 ± 2.40 a	18.20 ± 2.26	0.19 ± 0.01 cd	0.72 ± 0.07	0.56 ± 0.04 a	0.82 ± 0.03 abc	1.89 ± 0.18 b	nd
Im19	M	70.57 ± 2.17 b	22.07 ± 2.33	0.30 ± 0.04 abc	1.33 ± 0.33	0.89 ± 0.23 ab	1.29 ± 0.28 c	3.55 ± 0.44 ab	nd
cv. PMC	H	75.72 ± 1.03 ab	17.87 ± 1.63	0.40 ± 0.04 a	1.25 ± 0.13	1.53 ± 0.25 b	0.90 ± 0.20 abc	2.08 ± 0.10 ab	0.26 ± 0.13

Mean values (%) with ± St.Dev, and post hoc HSD Tukey test (lower case for significant differences at *p* ≤ 0.05); nd—not detected.

## Data Availability

All relevant data are contained within the article; the original contributions presented in the study are included in the article/Supplementary Material, further inquiries can be directed to the corresponding author.

## Supplementary Materials

The following supporting information can be downloaded at: https://www.mdpi.com/article/10.3390/plants13162338/s1.
