# Supplementary figures and images for: X-ray Microanalysis of Elemental Composition of Vitis sylvestris Pollen Grains
Source: Plants (Basel). 2024 Aug 22;13(16):2338. doi: 10.3390/plants13162338 (PMC11359539; doi:10.3390/plants13162338)

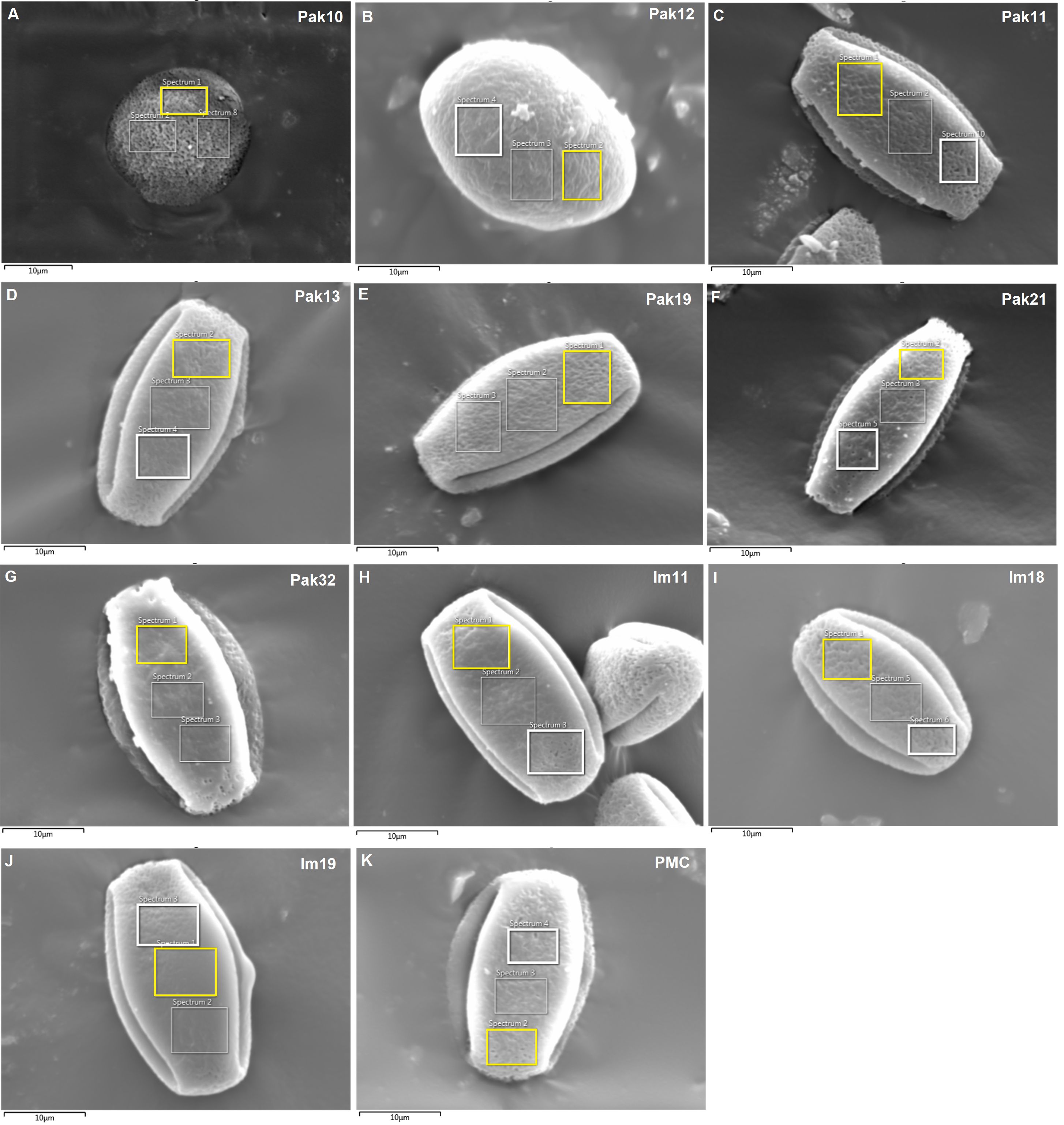

Supplement: Supplementary file 1 [file plants-13-02338-s001.zip › plants-3093787-supplementary.jpg]
